# Abnormal Neural Activation in Attention-Deficit/Hyperactivity Disorder: A Meta-Analysis of Functional Magnetic Resonance Imaging Studies

**DOI:** 10.1192/j.eurpsy.2024.195

**Published:** 2024-08-27

**Authors:** G. Zamora, C. Baten, A. M. Klassen, J. H. Shepherd, A. Catchpole, E. Davis, I. Dillsaver, C. E. Hunt, E. Johnson-Venegas, P. Hamilton, M. D. Sacchet, E. Woo, J. A. Miller, D. W. Hedges, C. H. Miller

**Affiliations:** ^1^Department of Psychology, California State University, Fresno, Fresno; ^2^Department of Psychology, Brigham Young University, Provo, United States; ^3^Department of Biological and Medical Psychology, University of Bergen, Bergen, Norway; ^4^Department of Psychiatry, Massachusetts General Hospital, Harvard Medical School, Boston; ^5^Department of Psychology, Palo Alto University, Palo Alto, United States

## Abstract

**Introduction:**

Attention-deficit/hyperactivity disorder (ADHD) is a highly prevalent psychiatric condition that frequently originates in early development and is associated with a variety of functional impairments. Despite a large functional neuroimaging literature on ADHD, our understanding of the neural basis of this disorder remains limited, and existing primary studies on the topic include somewhat divergent results.

**Objectives:**

The present meta-analysis aims to advance our understanding of the neural basis of ADHD by identifying the most statistically robust patterns of abnormal neural activation throughout the whole-brain in individuals diagnosed with ADHD compared to age-matched healthy controls.

**Methods:**

We conducted a meta-analysis of task-based functional magnetic resonance imaging (fMRI) activation studies of ADHD. This included, according to PRISMA guidelines, a comprehensive PubMed search and predetermined inclusion criteria as well as two independent coding teams who evaluated studies and included all task-based, whole-brain, fMRI activation studies that compared participants diagnosed with ADHD to age-matched healthy controls. We then performed multilevel kernel density analysis (MKDA) a well-established, whole-brain, voxelwise approach that quantitatively combines existing primary fMRI studies, with ensemble thresholding (p<0.05-0.0001) and multiple comparisons correction.

**Results:**

Participants diagnosed with ADHD (N=1,550), relative to age-matched healthy controls (N=1,340), exhibited statistically significant (p<0.05-0.0001; FWE-corrected) patterns of abnormal activation in multiple brains of the cerebral cortex and basal ganglia across a variety of cognitive control tasks.

**Conclusions:**

This study advances our understanding of the neural basis of ADHD and may aid in the development of new brain-based clinical interventions as well as diagnostic tools and treatment matching protocols for patients with ADHD. Future studies should also investigate the similarities and differences in neural signatures between ADHD and other highly comorbid psychiatric disorders.

**Disclosure of Interest:**

None Declared

